# Effectiveness of gonadotrophin-releasing hormone agonist therapy to improve the outcomes of intrauterine insemination in patients suffering from stage I-II endometriosis

**DOI:** 10.1080/07853890.2022.2071458

**Published:** 2022-05-10

**Authors:** Kemei Zhang, Shisi Huang, Haiyan Xu, Jiaou Zhang, Ensheng Wang, Yang Li, Changling Zhu, Jing Shu

**Affiliations:** Reproductive Medicine Center, Ningbo City First Hospital, Ningbo 315010, Zhejiang, China

**Keywords:** Endometriosis, intrauterine insemination, gonadotrophin releasing hormone agonist, clinical outcome, laparoscopy surgery

## Abstract

**Objective:**

To explore the role of postoperative gonadotrophin releasing hormone agonist (GnRH-a) therapy before treatment with intrauterine insemination (IUI) for infertile females with stage I-II endometriosis.

**Material and methods:**

Ninety-seven patients diagnosed with stage I-II endometriosis before IUI were enrolled in this study. The clinical pregnancy rate, cumulative pregnancy rate, live birth rate and newborn conditions were compared between the two groups with and without GnRH-a therapy.

**Results:**

The clinical pregnancy rate of IUI in the GnRH-a group was higher than that in the control group (15.29% vs. 11.82%, *p* = .035). By logistic regression analysis, patients treated with GnRH-a had a higher clinical pregnancy rate than those without (adjusted odds ratio (AOR) 23.190, 95% confidence interval (CI) 1.238–434.312). The live birth rate per IUI cycle in the GnRH-a group was also higher than in the controls (12.94% vs. 10%). However, the difference was not statistically significant (*p* = .311, AOR 4.844, 95% CI 0.229–102.320). The patients with GnRH-a therapy had a similar incidence of multiple pregnancy rate (0% vs. 0%), miscarriage rate (2.35% vs. 0.91%) and ectopic pregnancy rate (0% vs. 0.91%) as compared to the control group. The cumulative pregnancy rates were all higher in patients administered with GnRH-a than those without GnRH-a treatment in different cycles (one cycle: 17.07% vs 12.50%; two cycles: 29.27% vs 19.64%; three cycles: 31.71% vs 23.21%; ≥four cycles: 31.71% vs 23.21%), but the difference was not statistically significant. Notably, there was no more pregnancy after the third IUI cycle. The gestation weeks of delivery in the two groups were 39.09 ± 1.04 and 38.60 ± 1.17, respectively (*p* = .323). Nor was there difference in birth weight between the two groups (3236 ± 537 g vs 3435 ± 418 g, *p* = .360).

**Conclusions:**

The administration of GnRH-a in patients with stage I-II endometriosis could be beneficial to the outcomes of IUI. It is recommended that IUI should be discontinued after three failed attempts.

**KEY MESSAGES**
1. Endometriosis is a common cause of infertility, but the exact mechanism remains unclear.
2. The administration of GnRH-a before IUI treatment is beneficial for patients suffering from stage I-II endometriosis.
3. After three failed attempts, IUI should be stopped in patients with stage I-II endometriosis.

## Introduction

Endometriosis, a common gynecological disease and steroid-dependent disorder, is characterised by the presence of functional endometrial-type mucosa outside the uterine cavity [1,2]. It is estimated that 25–50% infertile patients have contracted endometriosis, and 30–50% women with endometriosis have trouble getting pregnant [3,4]. It has been widely accepted that infertility caused by endometriosis is due to the negative effects on oocyte quality, early embryo developmental potential and pelvic microenvironment for embryo implantation. In 1927, Sampson hypothesised the retrograde menstruation theory [5]. Since then, a great number of studies have been done to clarify the aetiology of endometriosis. As demonstrated before, endometriosis could induce female’s reproductive dysfunction by affecting the quality of oocytes and embryos [6,7], fallopian function [8] and embryo implantation [9]. It has been reported that the changes in follicular microenvironment of endometriosis patients is a contributing factor to infertility [10]. In a recent study, Lin et al. came to a conclusion that excessive reactive oxygen species induced pathological changes in ovary cumulus granulosa cell, which finally contributes to endometriosis-associated infertility [11]. However, it remains unclear about the exact mechanism of endometriosis-associated infertility, especially in the early stage of endometriosis [12,13]. Therefore, there is still controversy about the treatment strategy of patients with endometriosis. As an important public health problem, endometriosis related infertility deserves in-depth investigation.

Intrauterine insemination (IUI) is recommended for infertile couples with stage I-II endometriosis. As a first line treatment of assisted reproductive technology (ART), the advantages of IUI treatment are low cost and less invasion [14]. However, success rate of IUI is lower than *in vitro* fertilisation (IVF) and is affected by various factors, such as female age, duration of infertility and indication [15]. Especially, the pregnancy rate of IUI is very low in those patients with stage I-II endometriosis. It is therefore necessary to find chances to improve the clinical outcomes of IUI in infertile women with endometriosis.

Gonadotrophin releasing hormone agonist (GnRH-a) could be applied to lower the level of gonadotrophins which could inhibit the progression of endometriosis and prevent the formation of new lesions. In a recent meta-analysis, it has been concluded that both laparoscopy and GnRH-a alone could improve the clinical pregnancy outcomes of infertile women with endometriosis [16]. However, another investigation declared that there was no benefit for those patients with mild endometriosis to administer GnRH-a after laparoscopy surgeries [17].

In this study, our aim was to confirm the effectiveness of GnRH-a therapy by comparing the clinical pregnancy rate and live birth rate of IUI in patients suffering from stage I-II endometriosis with and without drug treatment after laparoscopy.

## Materials and methods

### Study design

We conducted a retrospective study by reviewing the clinical data of 195 cycles from 97 infertile females at the Reproductive Medicine Centre of Ningbo First Hospital in China between January 2015 and March 2021. All the subjects had undergone laparoscopy surgeries combined with hysteroscopy before IUI treatment. The overall pelvic environment and intrauterine condition were evaluated during the surgery. Meanwhile, tubal patency examination was performed to check the function of fallopian tubes. Inclusion criteria in our research included: stage I or stage II endometriosis with two patent fallopian tubes. Our study was approved by the Institutional Review Board of Ningbo First Hospital (No. 2021RS060). Written informed consents for admission, surgery, special drugs, discharge and follow-up were obtained from all the participants during the whole medical process. The informed consents for the administration of GnRH-a were obtained prior to the injection postoperatively. All the procedures were in accordance with the approved guidelines.

We followed up the patients until all surgical procedures, postoperative therapies with GnRH-a and IUI treatments were completed. If the participants got pregnant, we ended the follow-up lasted after they were delivered.

### Patients

Patients with endometriosis were diagnosed for the presence of endometriotic lesions during the laparoscopy surgery and were staged according to the revised American Society for Reproductive Medicine (ASRM) classification system [18]. All patients were scored by r-AFS (American Fertility Society) system during the surgery. Infertile women with stage I endometriosis (score 1–5 points, minimal) and stage II endometriosis (score 6–15 points, mild) were included in this study. All endometriotic lesions were excised or destroyed by bipolar coagulation during the surgery.

Exclusion criteria included ovulatory dysfunction (polycystic ovary syndrome, hyperprolactinaemia, thyroid dysfunction or luteinized unruptured follicle syndrome), abnormal implantation (submucosal myoma, adenomyosis, endometrial polyp or intrauterine adhesion), poor ovarian reserve and male factor infertility (semen volume <1.5 ml, total sperm number <39 million per ejaculate; sperm concentration <15*10^6^/ml; vitality <58% live; progressive motility <32%; total motility <40%; morphologically normal forms <4.0%) according to guidelines recommended by World Health Organization (WHO) [19].

### GnRH-a therapy

Forty-one patients received 3.6 mg goserelin (Silk Road Business Park, Macclesfield, Cheshire, SK10 2 NA UK) on day 2–3 after laparoscopy surgery were categorised as GnRH-a therapy group. All patients were informed about the potential side effects of GnRH-a therapy, including hot flushes, sexual hypoactivity, vaginal dryness, depression and slight but reversible bone loss. All these patients were subjected to GnRH-a treatment every 4 weeks for 1–3 cycles. Patients without GnRH-a therapy were considered as the controls.

### Follicle monitoring and ovulation induction

Follicle growth was monitored by transvaginal ultrasonography since day 10 in a natural cycle.

Ovarian stimulation protocols were as follows:

1. Clomiphene citrate (CC): 50–100 mg/day starting from day 3–5 for 5 days.
2. Letrozole (LE) 2.5–5.0 mg/day from day 3–5 for 5 days.
3. Human menopausal gonadotrophin (HMG) 37.5–75 IU/day starting from day 3–5 for a variable duration depending on the response.

When at least one mature follicle had a diameter of 18 mm or more, ovulation was triggered with intramuscular injection of urinary human chorionic gonadotrophin (hCG) (5000–10,000 IU), or hypodermic injection of recombinant human chorionic gonadotrophin alfa (Ovidrel, 0.25 mg), or hypodermic injection of Triptorelin (0.1 mg).

### Semen treatment

On the day of IUI, husband’s semen was collected by masturbation after abstinence for 3–7 days and prepared with two-layer density gradient centrifugation after liquefaction. The volume of washed semen sample used for insemination was 0.3–0.5 ml.

### Intrauterine insemination (IUI)

IUI was performed 36–40 h after injection by a gynaecologist in an operating room adjacent to the laboratory. After the operation, the women were advised to rest for at least 30 min. The luteal support was used routinely in all patients since the day of ovulation. It consisted of Duphaston (Dydrogesterone Tablets, 20 mg/day, Abbott, Netherlands) for 14 days. A blood test for hCG assay was performed 14 days after insemination to confirm whether pregnancy had occurred. If the patient got a positive hCG, ultrasound examination could be performed 3 weeks later to confirm foetal viability. Clinical pregnancy was confirmed when there was ultrasonographic evidence. The primary outcome was the clinical pregnancy rate calculated by dividing the numbers of clinical pregnant patients by the total number of patients who underwent IUI treatment. Other pregnancy outcomes included live birth rate, gestation weeks of delivery, preterm delivery rate and birth weight.

### Statistical analysis

Statistical analysis was performed using the Statistical Package for Social Sciences (SPSS, version 26.0). Data were presented as the means ± SD or number (%). The baseline differences between the two groups were analysed by Student’s *t* test. Pearson’s Chi-square test or Fisher’s test was used to compare the ratios between the groups. We also used logistic regression to model the clinical pregnancy rate and live birth rate with control group as the reference. A value of *p* less than .05 was considered statistically significant.

## Results

### General patient characteristics

The most common side effects of GnRH-a therapy included hot flash (7.32%), sexual hypoactivity (2.44%) and vaginal dryness (9.77%), which were mild and temporal. No other severe side effects were observed in all subjects. As shown in Table 1, more patients with stage II endometriosis were given GnRH-a therapy (75.86% vs 24.14%), while fewer patients with stage I were treated with GnRH-a (27.94% vs 72.06%). The r-AFS score of patients with or without GnRH-a therapy were 7.56 ± 3.81 and 2.88 ± 1.95, respectively. The differences were both significant (*p* = .000). There were no statistical differences in terms of female age, body mass index (BMI), Antimullerian hormone (AMH), basal hormones before laparoscopy surgery, duration of infertility, female education, unhealthy lifestyle and medical history between the two groups.

**Table 1.** Characteristics of endometriosis patients with or without GnRH-a therapy.

Characteristics		Endometriosis (I-II)		*p*-Value
		GnRH-a therapy (*n* = 41)	Control (*n* = 56)	
Female age (y)	Means ± SD	30.17 ± 2.54	30.20 ± 2.48	.960
BMI (kg/m^2^)	Means ± SD	20.69 ± 2.34	21.17 ± 2.60	.189
AMH (ng/ml)	Means ± SD	2.49 ± 0.80	3.52 ± 1.80	.262
Basal hormones (before surgery)	FSH (IU/L)	6.09 ± 1.73	6.41 ± 1.45	.583
	LH (IU/L)	4.76 ± 2.19	4.75 ± 2.84	.984
Duration of infertility (y)	Means ± SD	3.10 ± 1.54	2.90 ± 1.24	.344
Stage of endometriosis	Stage I, *n* = 68	19 (27.94)	49 (72.06)	.000*
	Stage II, *n* = 29	22 (75.86)	7 (24.14)	
r-AFS score	Means ± SD	7.56 ± 3.81	2.88 ± 1.95	.000*
Female education	<High school, *n* (%)	3 (7.32)	9 (16.07)	.491
	High school graduate, *n* (%)	2 (4.88)	4 (7.14)	
	Some college/associate degree, *n* (%)	14 (34.15)	12 (21.43)	
	Bachelor’s degree, *n* (%)	20 (48.78)	26 (46.43)	
	Master’s degree, *n* (%) 7	2 (4.88)	5 (8.93)	
	Doctor’s degree, *n* (%) 0	0	0	
Unhealthy lifestyle	Smoking, *n* (%)	0	0	–
	Alcoholism, *n* (%)	0	0	
Medical history	Diabetes, *n* (%)	0	0	–
	Hypertension, *n* (%)	0	0	–
	Autoimmune disease, *n* (%)	1 (2.44)	3 (5.36)	.844
	Myomas, *n* (%)	5 (12.20)	14 (25)	.116
	Labor history, *n* (%)	0	2 (3.57)	.507
	C-section, *n* (%)	0	0	–
	Gynecologic surgery history, *n* (%)	0	4 (7.14)	.135
	ART history, *n* (%)	9 (21.95)	6 (10.71)	.131

GnRH-a: gonadotrophin releasing hormone agonist; SD: standard deviation; BMI: body mass index; AMH: antimullerian hormone; C-section: caesarean section; r-AFS: revised American Fertility Society; ART: assistant reproductive technology.

Data were presented as mean ± SD or number (%). Student’s *t* test was performed to analyse baseline differences, while Chi-square test or Fisher’s exact test were performed to compare ratios between the two groups. *p < .05.

### IUI cycle treatment

As demonstrated in Table 2, there were 195 IUI cycles performed in the final analysis. In primary infertile patients, the rate of GnRH-a therapy group was higher than that in the control group (50.68% vs 49.32%, *p* = .000), while the ratio of the GnRH-a group was lower (21.28% vs 78.72%, *p* = .000) in the secondary infertile women. The day 2–3 serum FSH and LH levels of the GnRH-a group were both lower than those in the control group (FSH 5.81 ± 2.12 vs. 7.64 ± 1.91, *p* = .000; LH 3.52 ± 3.08 vs.5.12 ± 2.14, *p* = .003). Totally, 105 natural cycles and 90 ovarian stimulated cycles were performed in our study, respectively. There was no significant difference with respect to ratio of natural cycle to ovarian stimulated cycle (*p* = .060). Besides, there was no difference in terms of follicle number, total motile sperm count (TMSC) and side effects of IUI treatment between the two groups. However, larger endometrial thickness was observed in the patients administered with GnRH-a when compared with the control group (9.93 ± 2.09 mm vs 9.29 ± 1.75 mm, *p* = .022).

**Table 2.** Characteristic of IUI Cycle treatment with or without GnRH-a therapy.

Characteristics		Endometriosis (I-II)		*p*-Value
		GnRH-a therapy (*n* = 85)	Control (*n* = 110)	
Type of infertility	Primary, *n* = 148	75 (50.68)	73 (49.32)	.000*
	Secondary, *n* = 47	10 (21.28)	37 (78.72)	
Hormones on day 2–3 of IUI cycle	FSH (IU/L)	5.81 ± 2.12	7.64 ± 1.91	.000*
	LH (IU/L)	3.52 ± 3.08	5.12 ± 2.14	.003*
Type of ovulation	Natural cycles, *n* = 105	39 (45.88%)	66 (60%)	.060
	Ovarian stimulated cycles, *n* = 90	46 (54.12%)	44 (40%)	
No. of dominate follicles at the time of hCG injection (diameter ≥ 14 mm)	Means ± SD	1.14 ± 0.35	1.09 ± 0.29	.265
Endometrial thickness at the time of HCG injection (mm)	Means ± SD	9.93 ± 2.09	9.29 ± 1.75	.022*
TMSC (*10^6^)	Means ± SD	30.44 ± 18.60	29.05 ± 16.97	.848
Side effects	Vaginal bleeding, *n* (%)	4 (4.71)	5 (4.55)	.958
	Pelvic inflammation, *n* (%)	0	0	–
	OHSS, *n* (%)	0	0	–

IUI: intrauterine insemination; TMSC: total motile sperm count; OHSS: ovarian hyperstimulation syndrome.

Data were presented as mean ± SD or number (%). Student’s *t* test and Chi-square test were performed accordingly. *p < .05.

### Clinical pregnancy rate and pregnant outcomes

**Figure 1.**
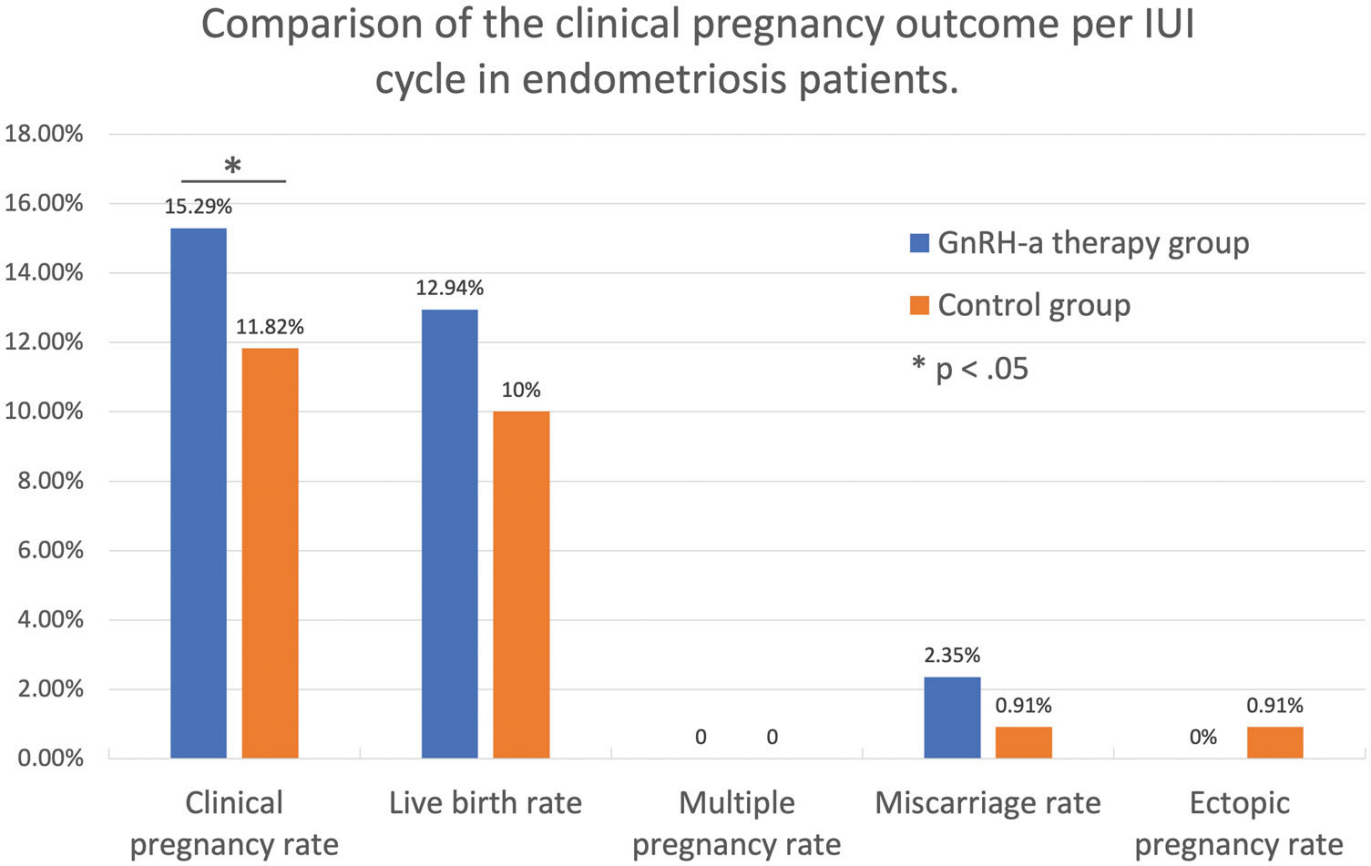
Comparison of the clinical pregnancy outcome per IUI cycle in endometriosis patients.

The clinical pregnancy rate of IUI treatment in GnRH-a group was higher than that in the control group (15.29% vs. 11.82%). The difference was statistically significant (*p* = .035) (Figure 1). As shown in Table 3, by logistic regression analysis, patients treated with GnRH-a had a higher clinical pregnancy rate compared to patients without GnRH-a therapy (adjusted odds ratio (AOR) 23.190, 95% confidence interval (CI) 1.238–434.312). The live birth rate per IUI cycle in the GnRH-a group was also higher than the control group (12.94% vs. 10%). However, the difference was not statistically significant (*p* = .311, AOR 4.844, 95% CI 0.229–102.320).

**Table 3.** Comparison of clinical outcomes by characteristics of patients and IUI cycle.

	Pregnancy rate (%)	*p*-Value	AOR	95%CI	Live birth rate (%)	*p*-Value	AOR	95%CI
GnRH-a therapy group (*n* = 85)	15.29	.035*	23.190	1.238–434.312	12.94	.311	4.844	0.229–102.320
Control group (*n* = 110)	11.82				10			
Cycle of GnRH-a therapy								
One cycle (*n* = 3)	66.67	.052	0.315	0.098–1.011	33.33	.380	0.587	0.179–1.929
Two cycles (*n* = 36)	16.67				13.88			
Three cycles (*n* = 46)	10.87				10.87			
Female age								
≤30y (*n* = 103)	13.59	.516	1.370	0.530–3.543	11.65	.755	1.171	0.434–3.154
>30y (*n* = 92)	13.04				10.87			
BMI								
<24 (*n* = 173)	13.87	.395	0.503	0.103–2.450	11.56	.679	0.717	0.149–3.453
≥24 (*n* = 22)	9.09				9.09			
Duration of infertility								
<3y (*n* = 82)	17.07	.311	0.617	0.242–1.571	13.41	.538	0.735	0.276–1.956
≥3y (*n* = 113)	10.62				9.73			
Type of infertility								
Primary (*n* = 148)	14.19	.557	0.716	0.234–2.187	11.49	.988	0.991	0.319–3.083
Secondary (*n* = 47)	10.64				10.64			
Stage of endometriosis								
Stage I (*n* = 134)	12.69	.974	1.019	0.335–3.099	10.45	.966	1.025	0.329–3.199
Stage II (*n* = 61)	14.75				13.11			
Cycle of IUI treatment								
First cycle (*n* = 98)	14.29	.574	0.847	0.474–1.513	12.24	.507	0.815	0.444–1.493
Second cycle (*n* = 62)	14.52				11.29			
Third cycle (*n* = 29)	10.34				10.34			
≥Fourth cycle (*n* = 6)	0				0			
Type of ovulation								
Natural cycle (*n* = 105)	14.29	.423	0.657	0.236–1.834	10.48	.973	1.018	0.353–2.934
Ovarian stimulated cycle (*n* = 90)	12.22				12.22			
Follicle number								
One follicle (*n* = 169)	12.42	.244	2.124	0.598–7.550	10.06	.180	2.352	0.674–8.210
Two follicles (*n* = 26)	19.23				19.23			
Endometrial thickness								
≤8 mm (*n* = 43)	18.60	.134	0.458	0.165–1.272	11.63	.872	0.910	0.290–2.855
>8 mm (*n* = 152)	11.84				11.18			
TMSC								
<20*10^6^/ml (*n* = 65)	13.85	.936	0.963	0.380–2.437	12.31	.801	0.884	0.337–2.314
≥20*10^6^/ml (130)	13.08				10.77			

AOR: adjusted odds ratio; CI: confidence interval. Data were presented as rate (%). Logistic regression was used to model the clinical pregnancy rate and delivery rate in GnRH-a therapy group with control group as the reference.

Overall, the patients with GnRH-a therapy had a similar incidence of multiple pregnancy rate (0% vs. 0%), miscarriage rate (2.35% vs. 0.91%) and ectopic pregnancy rate (0% vs. 0.91%) as compared to the control group (Figure 1). As in Table 4, the situation of live birth was also similar between the two groups. The gestation weeks of delivery in the two groups were 39.09 ± 1.04 and 38.60 ± 1.17, respectively (*p* = .323). Meanwhile, there was no difference in birth weight between patients with and without GnRH-a therapy (3236 ± 537 g vs 3435 ± 418 g, *p* = .360).

**Table 4.** Situation of live birth.

		Endometriosis (I-II)		*p*-Value
		GnRH-a therapy group (*n* = 11)	Control group (*n* = 11)	
Gestation weeks of delivery	Means ± SD	39.09 ± 1.04	38.60 ± 1.17	.323
Preterm delivery	*n* (%)	0 (0%)	1 (9.09%)	1.000
Delivery mode	Natural delivery, *n* = 7	3 (27.27%)	4 (36.36%)	1.000
	C-section, *n* = 15	8 (72.73%)	7 (63.64%)	
Birth weight (g)	Means ± SD	3236 ± 537	3435 ± 418	.360

C-section: caesarean section. Data were presented as mean ± SD or number (%). Student’s *t* test and Chi-square test were performed accordingly.

### Cumulative pregnancy rate

**Figure 2.**
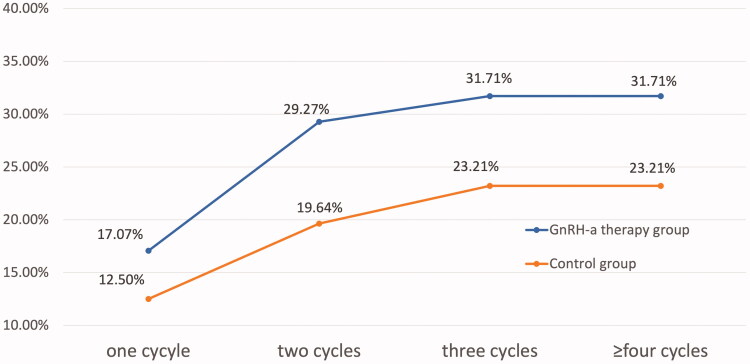
Comparison of cumulative pregnancy rate according to IUI cycles in endometriosis patients.

In our study, the cumulative pregnancy rate was assessed in 97 patients were up to 6 IUI cycles, which was the maximum number of cycles. As shown in Figure 2, the cumulative pregnancy rate was both higher in patients with administration of GnRH-a than those without GnRH-a treatment in different cycles (one cycle:17.07% vs 12.50%, *p* = .585; two cycles:29.27% vs 19.64%, *p* = .390; three cycles:31.71% vs 23.21%, *p* = .481; ≥four cycles:31.71% vs 23.21%, *p* = .481). But the difference was not statistically significant. As shown in table 3, the clinical pregnancy rate per different cycle was 14.29%, 14.52%, 10.34% and 0% respectively. Notably, there was no more pregnancy after the third cycle.

## Discussion

### Summary of findings

In our study, when comparing the general characteristics, including female age, BMI, duration of infertility, female education, unhealthy lifestyle, medical history, AMH and basal hormones before surgery in stage I-II endometriosis patients with and without GnRH-a treatment, there was no significant difference. More patients with stage II endometriosis were given GnRH-a therapy compared to those women with stage I, which was obviously reasonable. The serum FSH and LH levels on day 2–3 of menstruation in women with GnRH-a treatment were significantly lower than that in the control group (*p* = .000, *p* = .003). We consider the decreased serum FSH levels was due to the effect of GnRH-A therapy. The follicle number, TMSC and side effects of IUI treatment were similar between the two groups. However, the endometrial thickness in the GnRH-a therapy group was larger than that in the control group (*p* = .022), which might improve the clinical outcomes of IUI.

Our statistical analysis showed that the clinical pregnancy rate of IUI in stage I-II endometriosis infertile patients with GnRH-a therapy was higher than those without drug treatment. AOR analysed by logistic regression was 23.190, suggesting that the administration of GnRH-a could increase the chance of pregnancy significantly. No difference was observed in the condition of newborns between the two groups, which means the use of GnRH-a is safe for the offspring.

### Endometriosis and GnRH-a therapy

Treatment strategies of endometriosis-related infertility include laparoscopic surgery, medical treatments and assisted reproductive techniques. Emerging studies have been performed to identify a better intervention to improve the clinical outcomes in the field of endometriosis management [20,21]. Mounting evidence supports the effectiveness and acceptability of laparoscopic surgery [22–25]. However, the beneficial effect of GnRH-a therapy is still controversial.

GnRH-a is a hormone that could reduce the gonadotrophins level and improve the pelvic environment. Many studies have shown that the administration of GnRH-a in women with stage III-IV endometriosis for 3–6 cycles could reduce the recurrence of endometriosis and improve the pregnancy outcomes of infertile women before IVF/ICSI (intracytoplasmic sperm injection) [26]. However, whether the administration of GnRH-a in women with stage I-II endometriosis can be beneficial is still controversy.

Lin et al. reported that postoperative GnRH-a therapy was ineffective in improving reproductive outcomes in patients with both early (minimal or mild) and advanced (moderate and severe) endometriosis [17]. Bansal et al. also showed that there was no significant improvement in women undergoing IUI with the addition of GnRH-a for the suppression of mild endometriosis [27].

However, in our study, we found that the clinical pregnancy rate of IUI treatment could be elevated by GnRH-a therapy in patients with early endometriosis (stage I-II), which is consistent with a systematic review and network meta-analysis by Hodgson et al. [16]. As demonstrated before, high fertilisation rate could be expected if the secretion of FSH was suppressed [28]. Our study showed that the concentrations of FSH and LH were both lower in the GnRH-a group than those in the control group. Meanwhile, the endometrial thickness was also increased significantly by use of GnRH-a, which might be a result of the effectiveness of GnRH-a therapy in improving the endometrial responsiveness [29]. Based on our results, a benefit of postoperative GnRH-a treatment before IUI can be postulated.

Notably, the pregnancy rate of IUI treatment declined greatly after the second cycle in both GnRH-a group and control group. No more pregnancy occurred after the third cycle. Therefore, we recommend that IUI should be stopped in those patients after three failed attempts. We should review the cases and advise the patients to consider advanced ART treatments, such as IVF or ICSI.

### Strengths and limits

The strength of our study lies in its specific focus on the role of postoperative GnRH-a therapy before IUI treatment in infertile females with stage I-II endometriosis, which has not been targeted to explore thus far. The direct evidence comparing GnRH-a and blank control was very limited before. Meanwhile, all the patients were enrolled in our study without other causes of infertility to reduce the variation. All pregnant patients were followed up until delivery.

The limit of our research is that the number of subjects was limited. Therefore, further studies with a larger sample should be carried out to confirm the role of GnRH-a in IUI treatment for stage I-II endometriosis patients.

## Conclusion

In our study, the clinical pregnancy rate of IUI in stage I-II endometriosis patients treated with postoperative GnRH-a therapy was higher than in patients without, which needs to be further investigated in a larger sample size. The administration of GnRH-a in patients with stage I-II endometriosis seems to be beneficial to the outcomes of IUI. Notably, no more than three IUI cycles should be recommended for those infertile patients.

## Data Availability

All data will be shared on request to the corresponding author with permission of Ningbo First Hospital.
